# Supplementary material for: Temporal dynamics of climate change exposure and opportunities for global marine biodiversity
Source: Nat Commun. 2024 Jul 15;15:5836. doi: 10.1038/s41467-024-49736-6 (PMC11251284; doi:10.1038/s41467-024-49736-6)
Supplement: Supplementary file 1 — Supplementary Information [file 41467_2024_49736_MOESM1_ESM.pdf]

SUPPLEMENTARY INFORMATION

Temporal dynamics of climate change exposure and opportunities for global marine biodiversity

Andreas Schwarz Meyer[1], Alex L. Pigot[2], Cory Merow[3], Kristin Kaschner[4], Cristina Garilao[5], Kathleen Kesner-Reyes[6], Christopher H. Trisos[1,7]

[1]African Climate and Development Initiative, University of Cape Town, Cape Town, South Africa
[2]Centre for Biodiversity and Environment Research, Department of Genetics, Evolution and Environment, University College London, London, UK
[3]Department of Ecology and Evolutionary Biology and Eversource Energy Center, University of Connecticut, Storrs, CT, USA
[4]GEOMAR Helmholtz-Centre for Ocean Research, Kiel, Germany
[5]Department of Biometry and Environmental Systems Analysis, Albert-Ludwigs University, Freiburg im Breisgau, Germany
[6]Quantitative Aquatics, Los Baños, Philippines
[7]African Synthesis Centre for Climate Change Environment and Development (ASCEND), University of Cape Town, Cape Town, South Africa

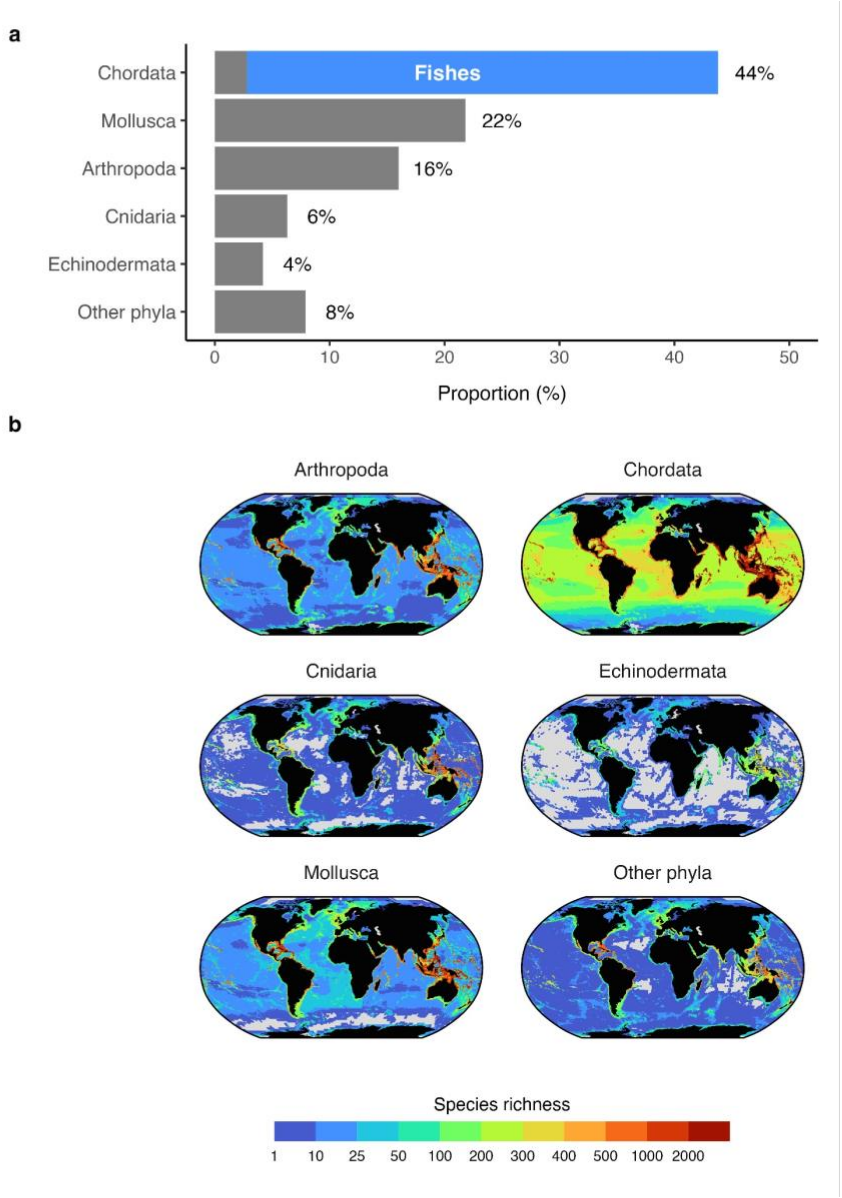

Supplementary Fig. 1 | a, Proportion of species among the five phyla with more species in the dataset used in this study. **b,** Species richness per phyla.

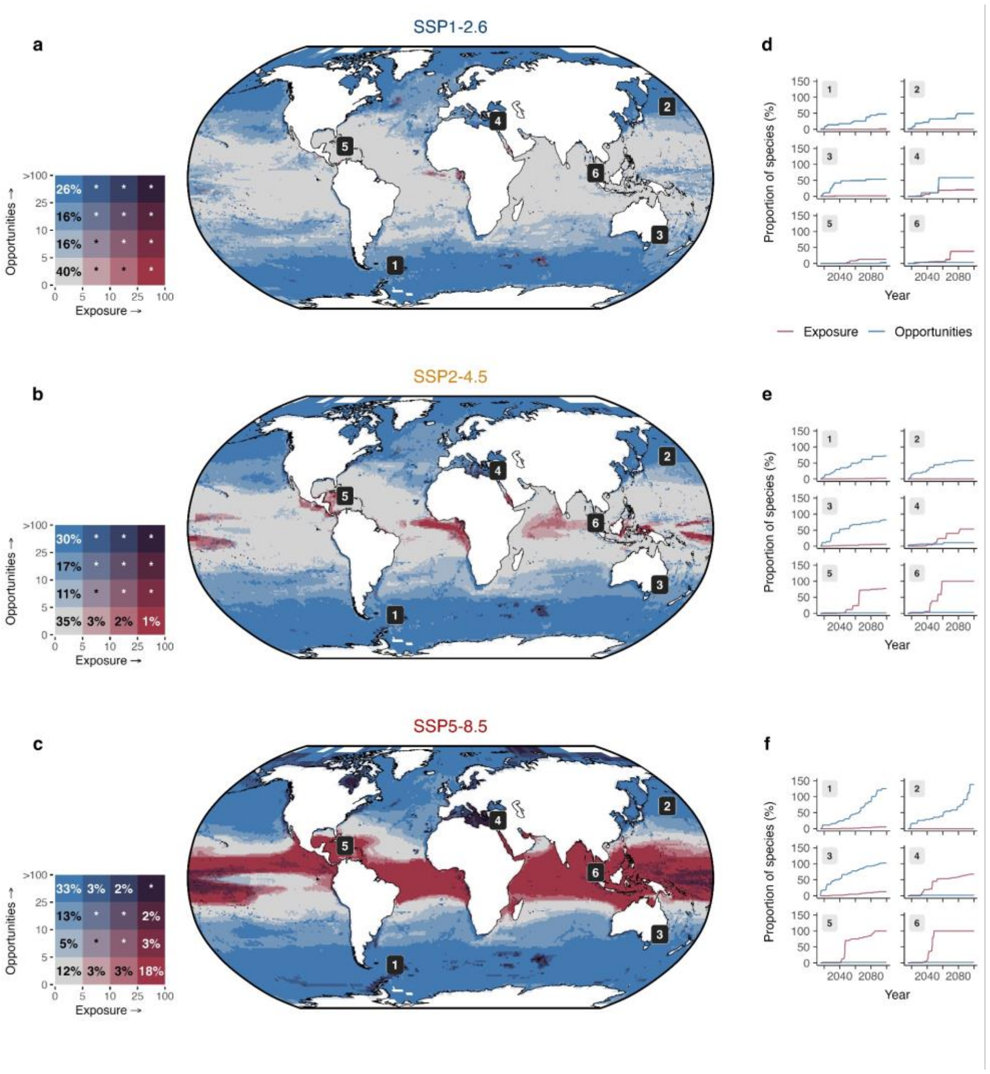

Supplementary Fig. 2 | The projected magnitude of thermal exposure and opportunity for marine biodiversity show distinct spatial patterns. Results were obtained using a dispersal rate of 50 km $year^{-1}$. **a-c**, Bivariate maps showing the proportion of species exposed (x-axis) and opportunities created (y-axis) as a percentage of local species richness across low (SSP1-2.6), intermediate (SSP2-4.5), and high (SSP5-8.5) emission scenarios. The percentages inside the key indicate the proportion of assemblages (that is, species in 100 km grid cells) within each bivariate bin. Asterisks indicate values below 1%. Lowering emissions from SSP5-8.5 (**c**) to SSP1-2.6 (**a**) has a greater impact on reducing exposure than on opportunities. **d-f**, Examples of exposure and opportunity profiles for local assemblages for each emission scenario. Profiles correspond to the scenario represented on the map adjacent to the plots. Exposure was estimated only for native species. Opportunities are concentrated in temperate and polar regions, while exposure occurs mostly in the tropics. The profiles show how opportunities accumulate more gradually (sites 1-4) while exposure can be abrupt (sites 5 and 6). Most regions show either high opportunity or high exposure, although regions such as the Mediterranean (4) can show both. The figure shows the median value across 9 climate models.

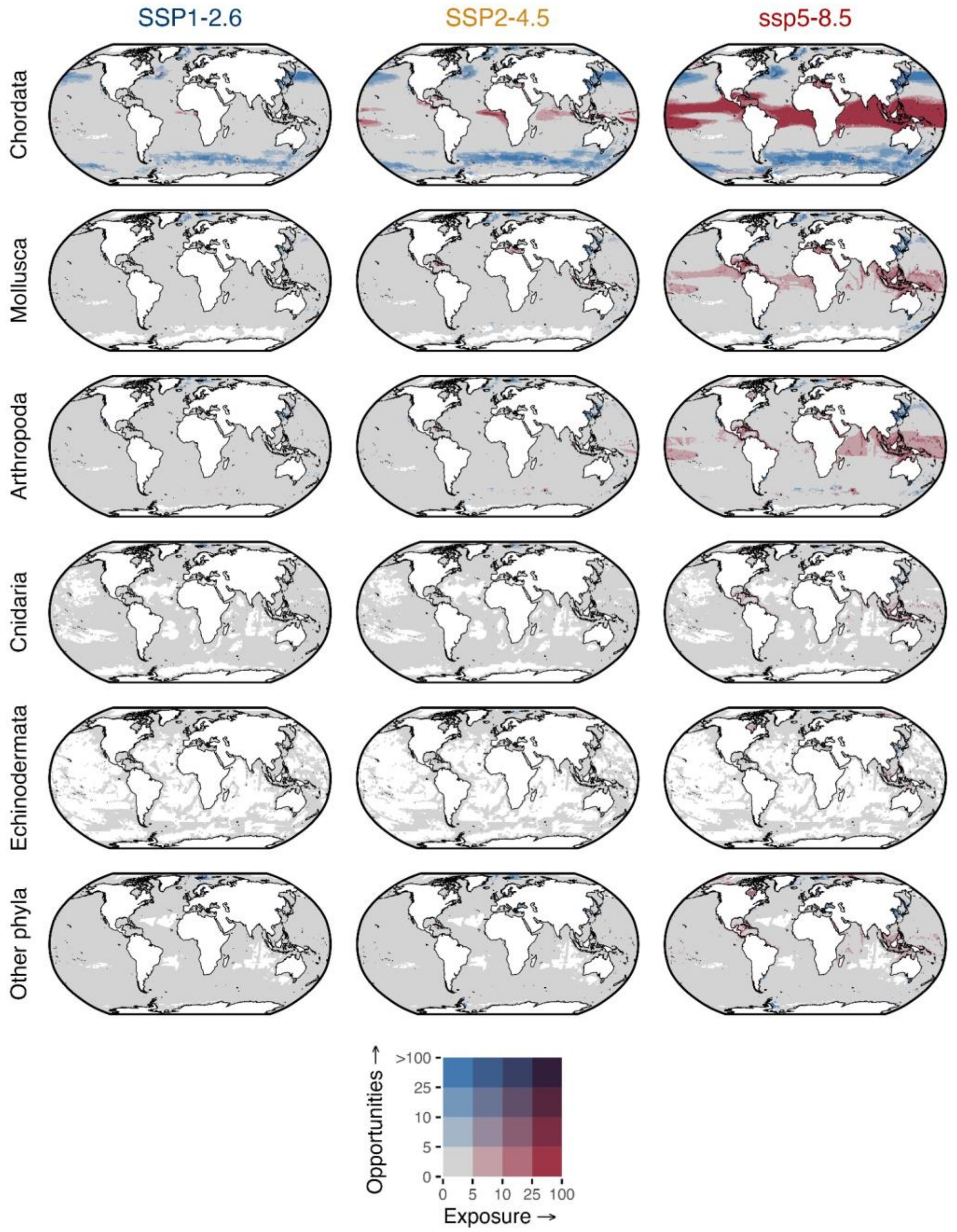

Supplementary Fig. 3 | Projected magnitude of thermal exposure and opportunity for each phylum as a percentage of total species richness in a grid cell. Results were obtained using a dispersal rate of 10 km year^{-1}. Exposure is the number of species in a phyla exposed as a percentage of all species richness in a grid cell. Opportunity is the number of opportunities that arose for a phyla as a percentage of all species richness in a grid cell. White grid cells show where species richness for a phylum is zero.

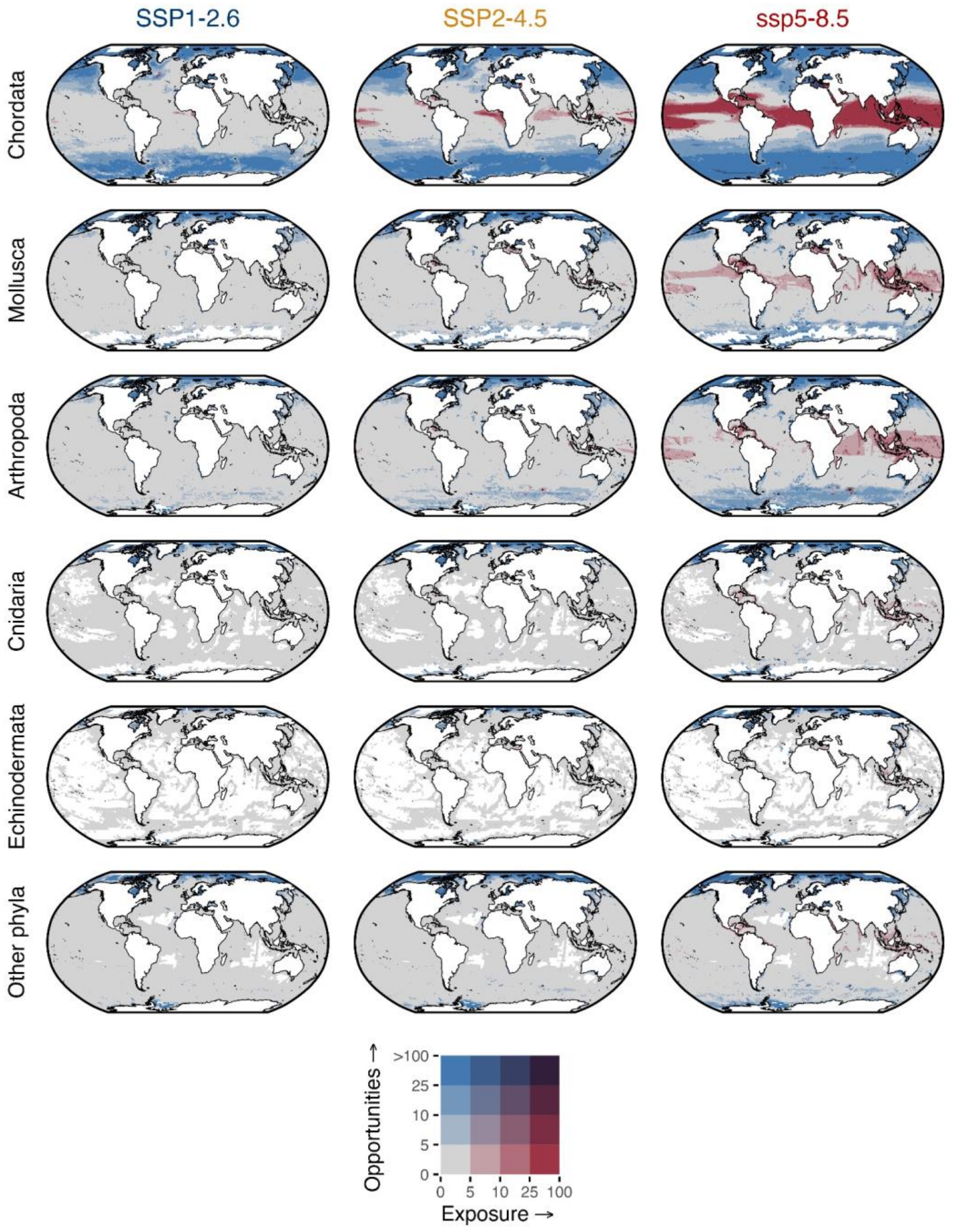

Supplementary Fig. 4 | Projected magnitude of thermal exposure and opportunity for each phylum as a percentage of total species richness in a grid cell. Results were obtained using a dispersal rate of 50 km year^{-1}. Exposure is the number of species in a phyla exposed as a percentage of all species richness in a grid cell. Opportunity is the number of opportunities that arose for a phyla as a percentage of all species richness in a grid cell. White grid cells show where species richness for a phylum is zero.

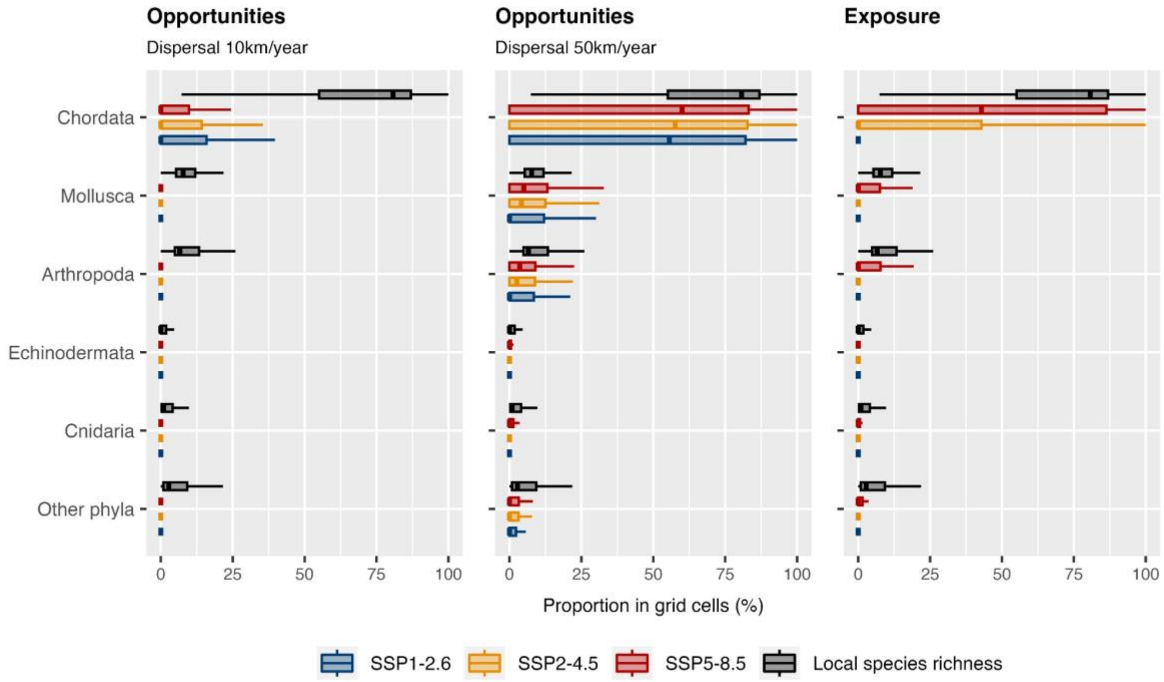

Supplementary Fig. 5 | The distribution across phyla of opportunities and exposure in grid cells. Boxplots summarise data from all grid cells (i.e. assemblages) analysed. Black boxplots show the proportion of species richness in a grid cell comprised of species from a given phyla. The proportion of all species opportunities or exposures in a grid cell comprised of species from a given phyla is shown by blue (SSP1-2.6), yellow (SSP2-4.5), and red (SSP5-8.5) boxplots. **a,** Data from opportunities under a 10 km year^{-1} dispersal rate. **b,** Data from opportunities under a 50 km year^{-1} dispersal rate. **c,** Data from exposure.

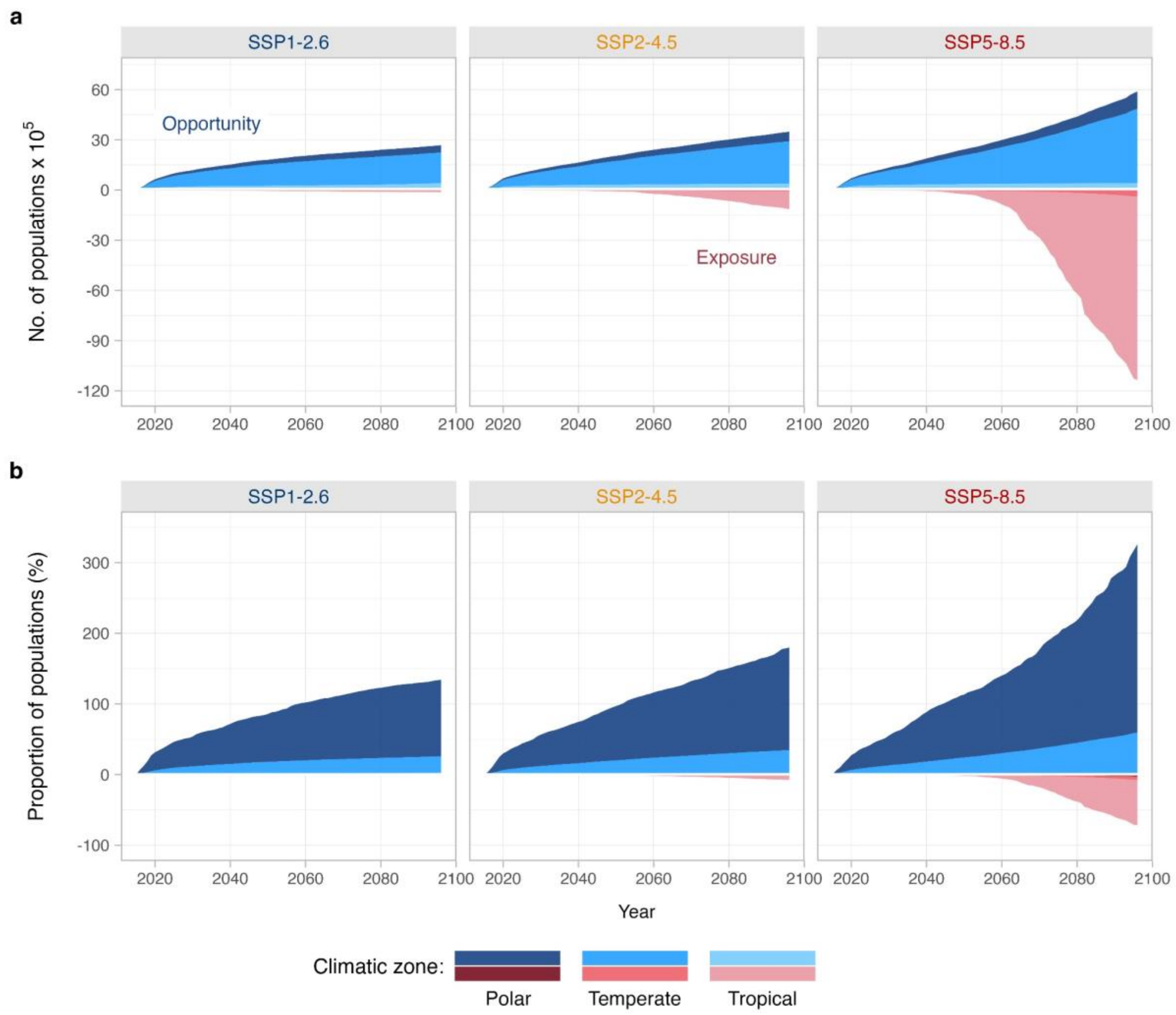

Supplementary Fig. 6 | Global profiles of thermal exposure and opportunities for marine biodiversity show earlier emergence of opportunities. Results from a 50 km year^{-1} dispersal rate. **a**, The cumulative number of populations exposed (red) and opportunities (blue) over time across three emission scenarios. A population is defined as a species occurrence in an assemblage (i.e. grid cell). The different colour shades indicate the climatic zone where exposure and opportunity are projected (polar, temperate, or tropical zones). **b**, Same data as in **a**, but with exposure and opportunity shown as a proportion of the current number of populations within each climatic zone. Opportunities arise early in the century and follow a similar trajectory across scenarios until 2040. Exposure starts later and is significantly lower under SSP1-2.6 and SSP2-4.5 when compared to SSP5-8.5. The panels show the median value across 9 climate models.

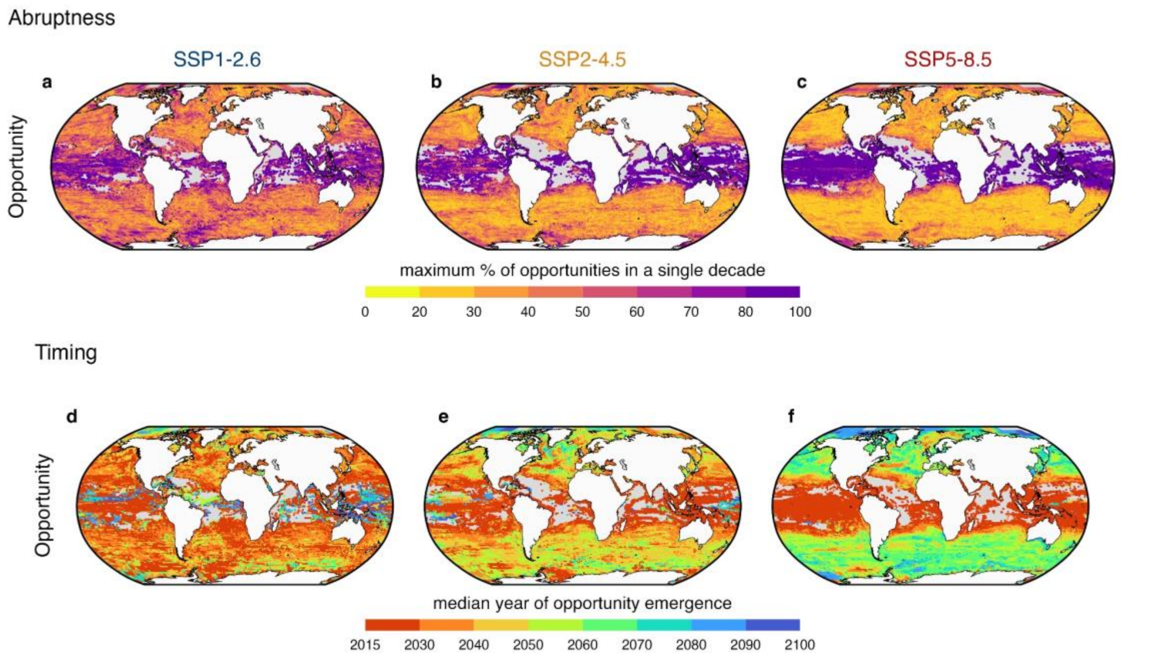

Supplementary Fig. 7 | Global variation in the abruptness and timing of opportunity. Results from a 50 km year^{-1} dispersal rate. Each column shows results from a different emission scenario. **a-c**, Abruptness. **d-f**, Timing. Opportunities arise earlier and more abruptly under SSP1-2.6 when compared to SSP5-8.5 and SSP2-4.5. The maps show the metrics calculated from the median across 9 climate models. Only communities with more than five opportunities are shown.

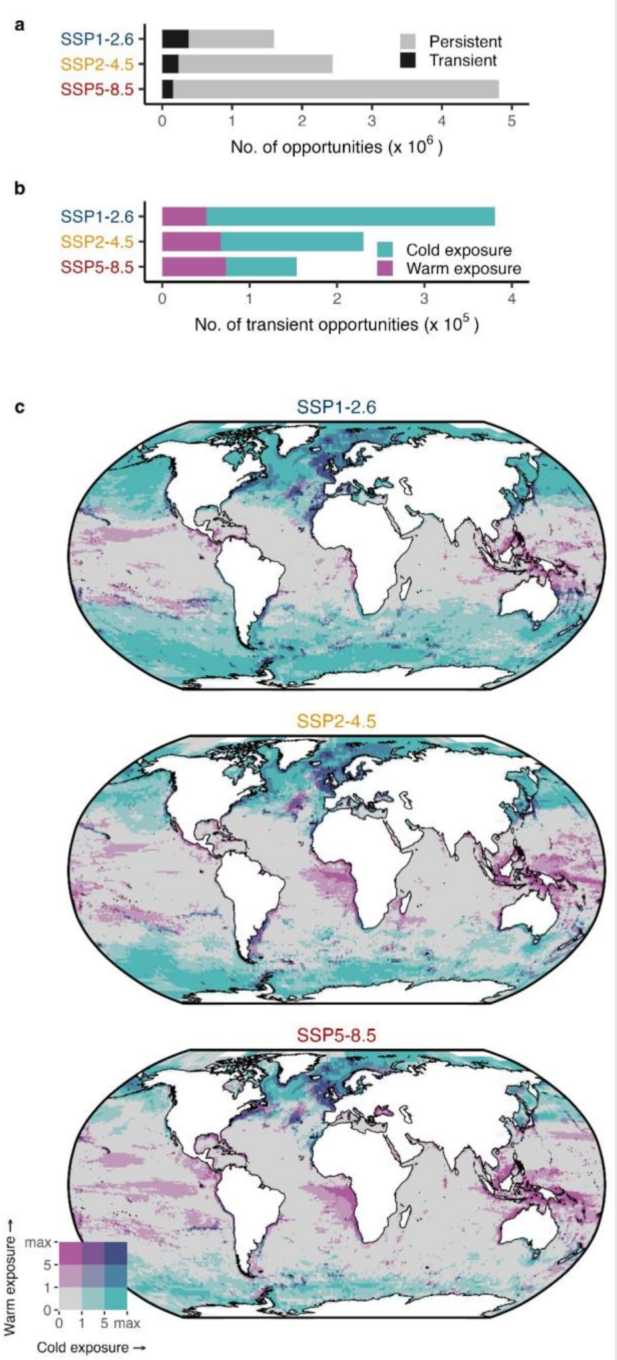

Supplementary Fig. 8 | Frequency and distribution of persistent and transient opportunities. Results from a 50 km year^{-1} dispersal rate. **a**, Frequency of persistent and transient thermal opportunities. **b**, Frequency of transient opportunities closed by exposure to temperatures above (warm exposure) and below (cold exposure) the realised thermal niche limits of the species. **c**, Bivariate maps showing the geographical distribution of the median number of warm- and cold-exposed opportunities for three emission scenarios. The key indicates the number of transient opportunities. All figures show the mean value across 9 climate models.

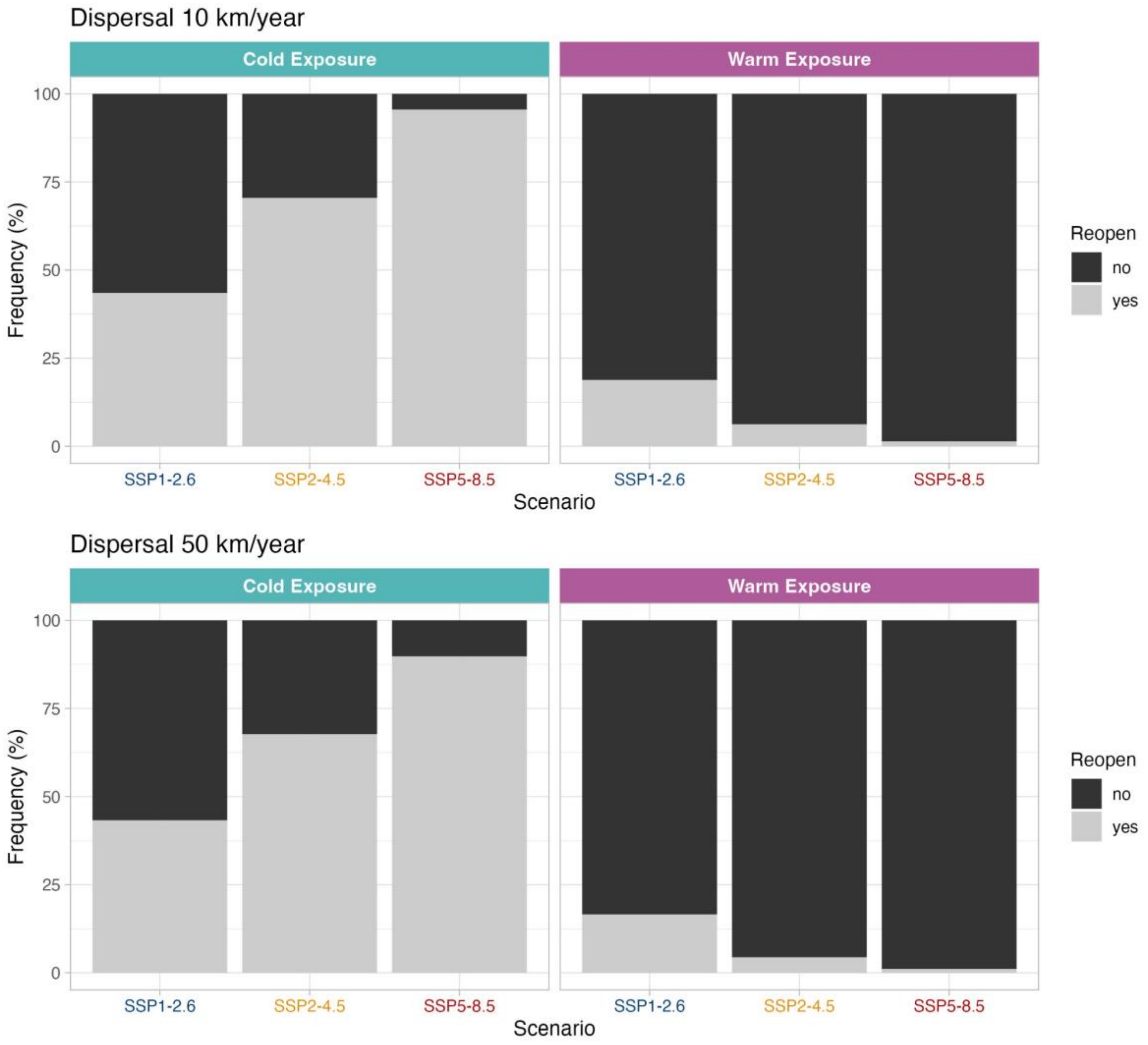

Supplementary Fig. 9 | Reopen frequency for transient opportunities closed due to cold (left) and warm exposure (right). Top row shows results for a 10 km year^{-1} dispersal rate. Bottom row shows results for a 50 km year^{-1} dispersal rate.

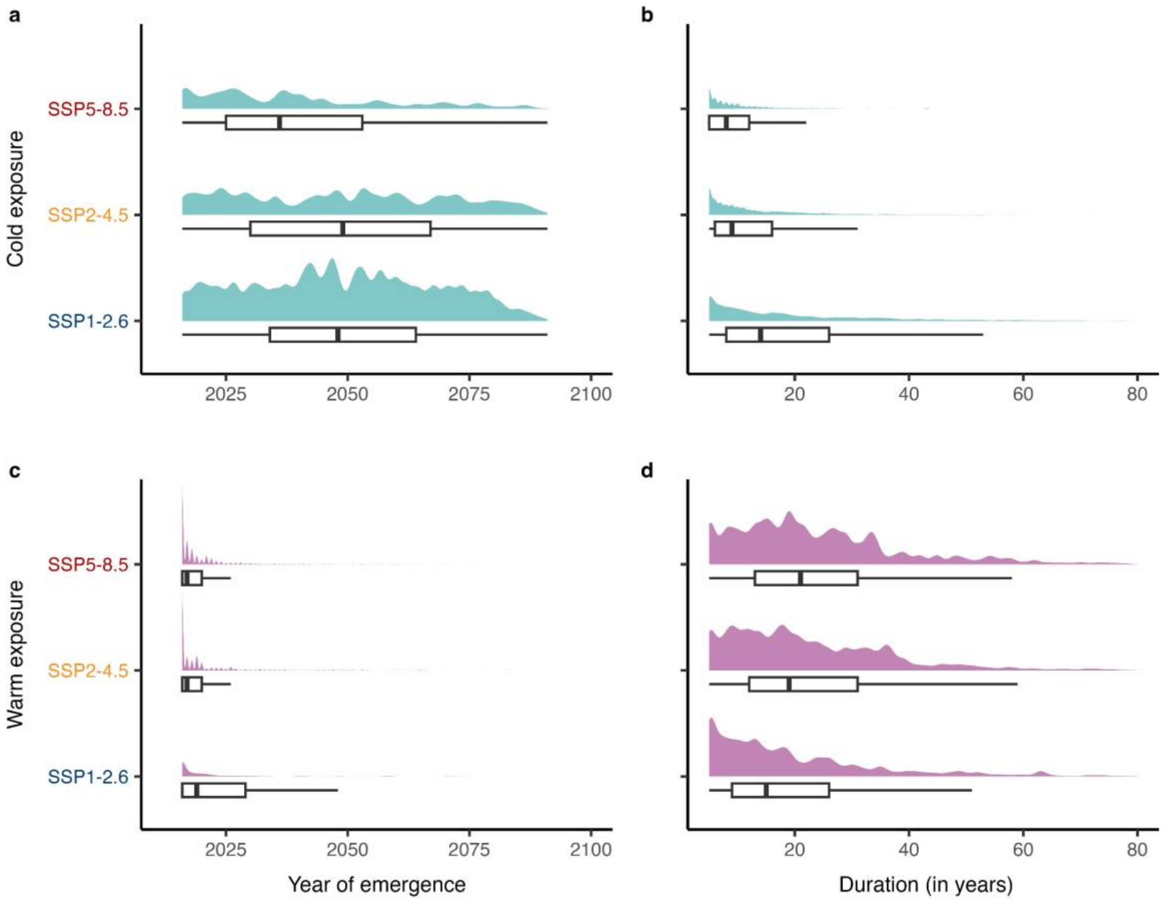

Supplementary Fig. 10 | Year of emergence and duration of transient opportunities. Results from a 50 km year^{-1} dispersal rate. Each figure includes both a density plot scaled proportionally to the number of observations and a boxplot for three emission scenarios. **a**, Year of emergence and **b**, duration of transient opportunities closed due to exposure to cold temperatures. **c**, Year of emergence and **d**, duration of transient opportunities closed due to exposure to warm temperatures. Transient opportunities closed due to warm exposure generally arise earlier and last longer than those closed due to cold exposure. All figures show the mean value across 9 climate models. Boxplots display the median (centre) and the 25th and 75th percentiles (lower and upper bounds of the box). The upper and lower whiskers extend to ±1.5 times the interquartile range. Outliers beyond the whiskers are not shown.

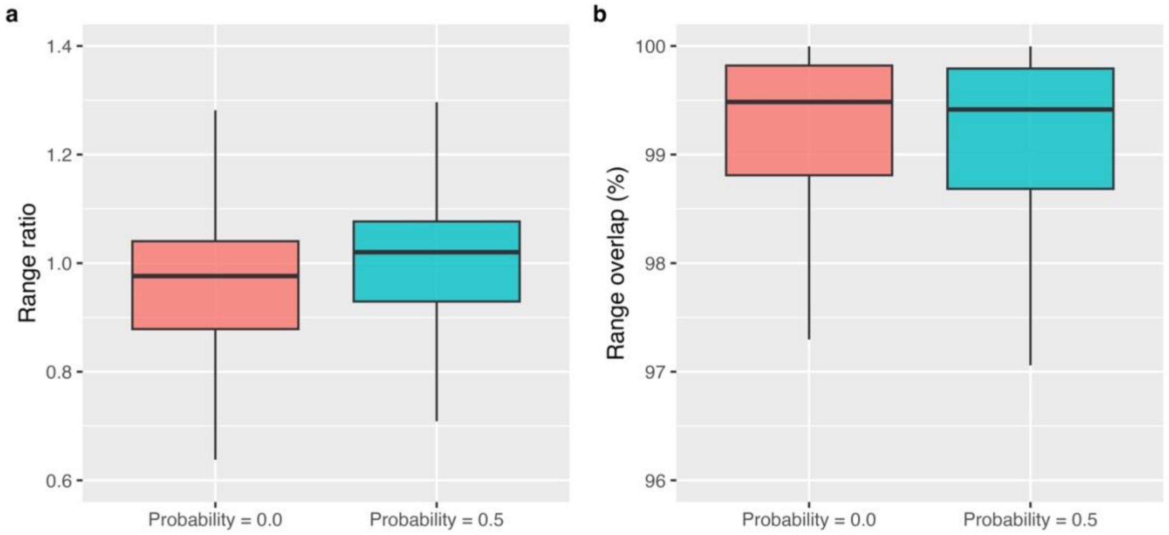

Supplementary Fig. 11 | Comparison between AquaMaps and IUCN range size and overlap. a, Comparison of range size estimates from IUCN and AquaMaps (N = 2,510 species). Range sizes were compared by dividing the logarithm of the range size obtained from AquaMaps by the logarithm of the range size from IUCN. If the result is > 1, the range size from IUCN is larger than the range size from AquaMaps. b, Percentage of spatial overlap between geographic ranges estimates from IUCN and AquaMaps. The overlap was calculated by quantifying the shared grid cells between the IUCN and AquaMaps and then dividing the number of shared grid cells by the number of unique grid cells from both datasets. Each boxplot shows results comparing IUCN estimates with AquaMaps estimates obtained using a probability of occurrence of 0.0 (orange) and 0.5 (green).

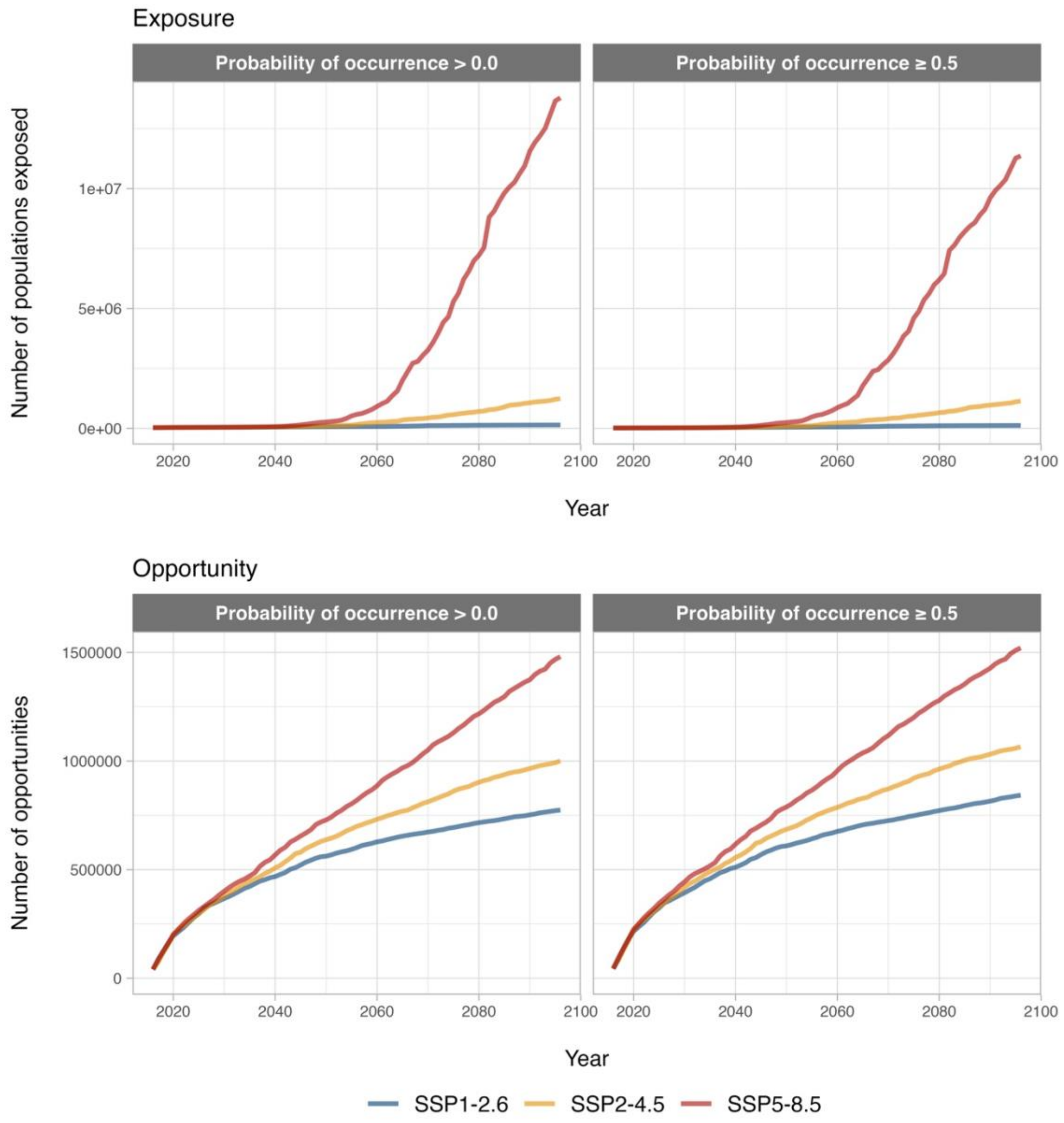

Supplementary Fig. 12 | Cumulative exposure and opportunity estimates obtained using two occurrence probability thresholds from AquaMaps. Exposure (top) and opportunity (bottom) estimates were obtained using a relative probability of 0.0 (left) and 0.5 (right) and a 10 km year-1 dispersal rate. Results obtained using both thresholds were similar for all scenarios, except for exposure under SSP5-8.5. For this scenario, the magnitude estimated using the 0.0 threshold was 21% higher than the magnitude estimated using the 0.5 threshold. This difference can be attributed to the differences in species range size obtained using each threshold, as a 0.0 threshold predicts a large range, resulting in a higher number of populations that can be exposed.

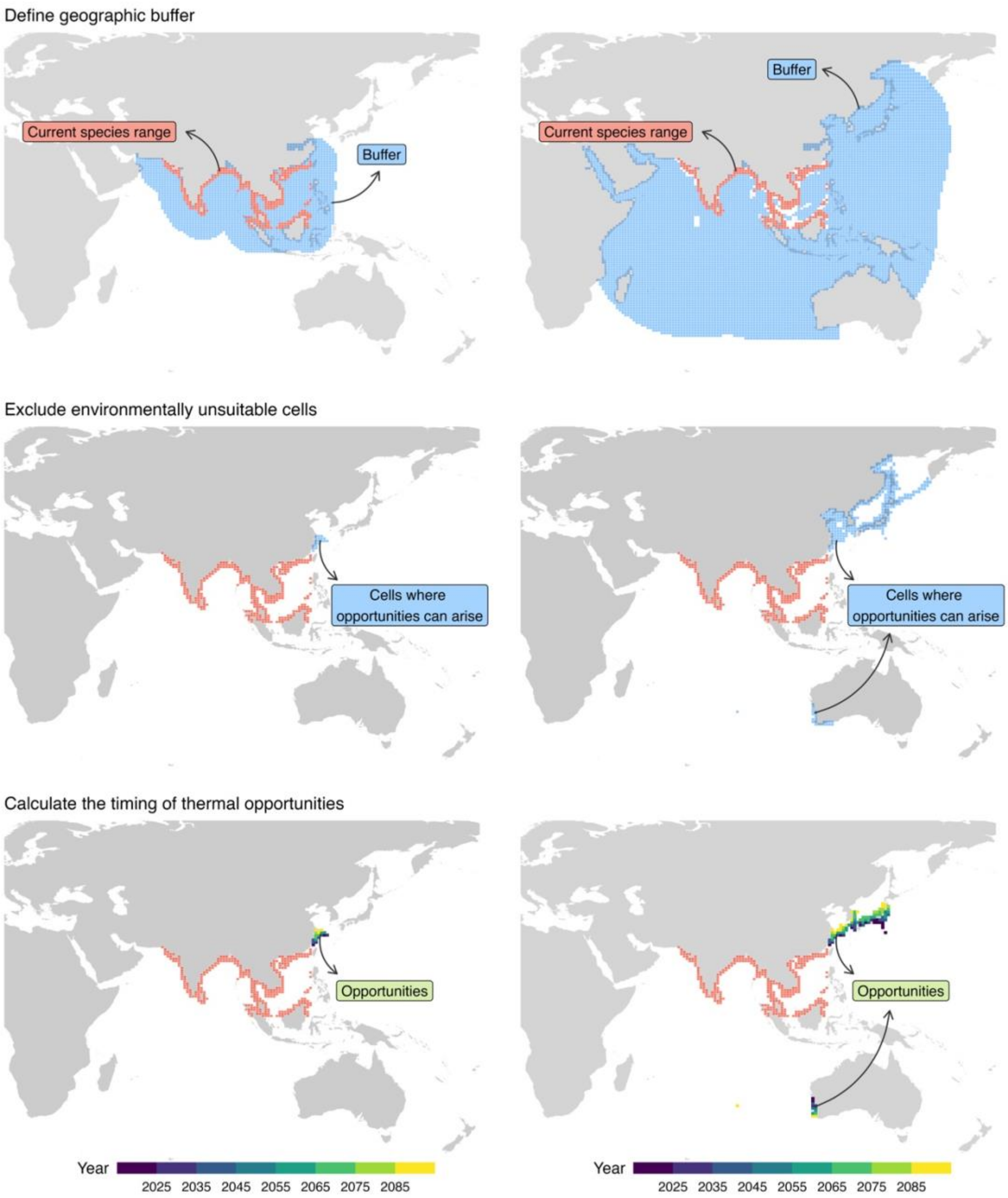

Supplementary Fig. 13 | Workflow used to constrain and estimate opportunities. Example based on the distribution of the bronze croaker *Otolithoides biauritus* using sea surface temperature projections from the model CNRM-CM6-1. The figure shows estimates obtained using two buffer sizes: 860 km (left) and 4300 km (right). First, we defined a buffer around the distribution of the species (top). Second, we excluded from the buffer environmental unsuitable cells, which are cells that had either unsuitable depth for the species or cells in which temperature fell within the niche limits of the species but were predicted as unsuitable by AquaMaps (middle). Finally, we estimated when opportunities will arise (bottom).

Supplementary Table 1 | List of references from the climate models used in the study.

Model	Scenario	Reference
CanESM5	SSP1-2.6	Swart, Neil Cameron; Cole, Jason N.S.; Kharin, Viatcheslav V.; Lazare, Mike; Scinocca, John F.; Gillett, Nathan P.; Anstey, James; Arora, Vivek; Christian, James R.; Jiao, Yanjun; Lee, Warren G.; Majaess, Fouad; Saenko, Oleg A.; Seiler, Christian; Seinen, Clint; Shao, Andrew; Solheim, Larry; von Salzen, Knut; Yang, Duo; Winter, Barbara; Sigmond, Michael (2019). CCCma CanESM5 model output prepared for CMIP6 ScenarioMIP SSP1-2.6. Earth System Grid Federation. doi: https://doi.org/10.22033/ESGF/CMIP6.3683.
CanESM5	SSP2-4.5	Swart, Neil Cameron; Cole, Jason N.S.; Kharin, Viatcheslav V.; Lazare, Mike; Scinocca, John F.; Gillett, Nathan P.; Anstey, James; Arora, Vivek; Christian, James R.; Jiao, Yanjun; Lee, Warren G.; Majaess, Fouad; Saenko, Oleg A.; Seiler, Christian; Seinen, Clint; Shao, Andrew; Solheim, Larry; von Salzen, Knut; Yang, Duo; Winter, Barbara; Sigmond, Michael (2019). CCCma CanESM5 model output prepared for CMIP6 ScenarioMIP SSP2-4.5. Earth System Grid Federation. doi: https://doi.org/10.22033/ESGF/CMIP6.3685.
CanESM5	SSP5-8.5	Swart, Neil Cameron; Cole, Jason N.S.; Kharin, Viatcheslav V.; Lazare, Mike; Scinocca, John F.; Gillett, Nathan P.; Anstey, James; Arora, Vivek; Christian, James R.; Jiao, Yanjun; Lee, Warren G.; Majaess, Fouad; Saenko, Oleg A.; Seiler, Christian; Seinen, Clint; Shao, Andrew; Solheim, Larry; von Salzen, Knut; Yang, Duo; Winter, Barbara; Sigmond, Michael (2019). CCCma CanESM5 model output prepared for CMIP6 ScenarioMIP SSP5-8.5. Earth System Grid Federation. doi: https://doi.org/10.22033/ESGF/CMIP6.3696.
CNRM-CM6-1	SSP1-2.6	Voldoire, Aurore (2019). CNRM-CERFACS CNRM-CM6-1 model output prepared for CMIP6 ScenarioMIP SSP1-2.6. Earth System Grid Federation. doi: https://doi.org/10.22033/ESGF/CMIP6.4184.
CNRM-CM6-1	SSP2-4.5	Voldoire, Aurore (2019). CNRM-CERFACS CNRM-CM6-1 model output prepared for CMIP6 ScenarioMIP SSP2-4.5. Earth System Grid Federation. doi: https://doi.org/10.22033/ESGF/CMIP6.4189.
CNRM-CM6-1	SSP5-8.5	Voldoire, Aurore (2019). CNRM-CERFACS CNRM-CM6-1 model output prepared for CMIP6 ScenarioMIP SSP5-8.5. Earth System Grid Federation. doi: https://doi.org/10.22033/ESGF/CMIP6.4224.
CNRM-ESM2-1	SSP1-2.6	Voldoire, Aurore (2019). CNRM-CERFACS CNRM-ESM2-1 model output prepared for CMIP6 ScenarioMIP SSP1-2.6. Earth System Grid Federation. doi: https://doi.org/10.22033/ESGF/CMIP6.4186.
CNRM-ESM2-1	SSP2-4.5	Voldoire, Aurore (2019). CNRM-CERFACS CNRM-ESM2-1 model output prepared for CMIP6 ScenarioMIP SSP2-4.5. Earth System Grid Federation. doi: https://doi.org/10.22033/ESGF/CMIP6.4191.
CNRM-ESM2-1	SSP5-8.5	Voldoire, Aurore (2019). CNRM-CERFACS CNRM-ESM2-1 model output prepared for CMIP6 ScenarioMIP SSP5-8.5. Earth System Grid Federation. doi: https://doi.org/10.22033/ESGF/CMIP6.4226.

MPI-ESM1-2-HR	SSP1-2.6	Schupfner, Martin; Wieners, Karl-Hermann; Wachsmann, Fabian; Steger, Christian; Bittner, Matthias; Jungclaus, Johann; Früh, Barbara; Pankatz, Klaus; Giorgetta, Marco; Reick, Christian; Legutke, Stephanie; Esch, Monika; Gayler, Veronika; Haak, Helmuth; de Vrese, Philipp; Raddatz, Thomas; Mauritsen, Thorsten; von Storch, Jin-Song; Behrens, Jörg; Brovkin, Victor; Claussen, Martin; Crueger, Traute; Fast, Irina; Fiedler, Stephanie; Hagemann, Stefan; Hohenegger, Cathy; Jahns, Thomas; Kloster, Silvia; Kinne, Stefan; Lasslop, Gitta; Kornblueh, Luis; Marotzke, Jochem; Matei, Daniela; Meraner, Katharina; Mikolajewicz, Uwe; Modali, Kameswarrao; Müller, Wolfgang; Nabel, Julia; Notz, Dirk; Peters-von Gehlen, Karsten; Pincus, Robert; Pohlmann, Holger; Pongratz, Julia; Rast, Sebastian; Schmidt, Hauke; Schnur, Reiner; Schulzweida, Uwe; Six, Katharina; Stevens, Bjorn; Voigt, Aiko; Roeckner, Erich (2019). DKRZ MPI-ESM1.2-HR model output prepared for CMIP6 ScenarioMIP SSP1-2.6. Earth System Grid Federation. doi: https://doi.org/10.22033/ESGF/CMIP6.4397.
MPI-ESM1-2-HR	SSP2-4.5	Schupfner, Martin; Wieners, Karl-Hermann; Wachsmann, Fabian; Steger, Christian; Bittner, Matthias; Jungclaus, Johann; Früh, Barbara; Pankatz, Klaus; Giorgetta, Marco; Reick, Christian; Legutke, Stephanie; Esch, Monika; Gayler, Veronika; Haak, Helmuth; de Vrese, Philipp; Raddatz, Thomas; Mauritsen, Thorsten; von Storch, Jin-Song; Behrens, Jörg; Brovkin, Victor; Claussen, Martin; Crueger, Traute; Fast, Irina; Fiedler, Stephanie; Hagemann, Stefan; Hohenegger, Cathy; Jahns, Thomas; Kloster, Silvia; Kinne, Stefan; Lasslop, Gitta; Kornblueh, Luis; Marotzke, Jochem; Matei, Daniela; Meraner, Katharina; Mikolajewicz, Uwe; Modali, Kameswarrao; Müller, Wolfgang; Nabel, Julia; Notz, Dirk; Peters-von Gehlen, Karsten; Pincus, Robert; Pohlmann, Holger; Pongratz, Julia; Rast, Sebastian; Schmidt, Hauke; Schnur, Reiner; Schulzweida, Uwe; Six, Katharina; Stevens, Bjorn; Voigt, Aiko; Roeckner, Erich (2019). DKRZ MPI-ESM1.2-HR model output prepared for CMIP6 ScenarioMIP SSP2-4.5. Earth System Grid Federation. doi: https://doi.org/10.22033/ESGF/CMIP6.4398.
MPI-ESM1-2-HR	SSP5-8.5	Schupfner, Martin; Wieners, Karl-Hermann; Wachsmann, Fabian; Steger, Christian; Bittner, Matthias; Jungclaus, Johann; Früh, Barbara; Pankatz, Klaus; Giorgetta, Marco; Reick, Christian; Legutke, Stephanie; Esch, Monika; Gayler, Veronika; Haak, Helmuth; de Vrese, Philipp; Raddatz, Thomas; Mauritsen, Thorsten; von Storch, Jin-Song; Behrens, Jörg; Brovkin, Victor; Claussen, Martin; Crueger, Traute; Fast, Irina; Fiedler, Stephanie; Hagemann, Stefan; Hohenegger, Cathy; Jahns, Thomas; Kloster, Silvia; Kinne, Stefan; Lasslop, Gitta; Kornblueh, Luis; Marotzke, Jochem; Matei, Daniela; Meraner, Katharina; Mikolajewicz, Uwe; Modali, Kameswarrao; Müller, Wolfgang; Nabel, Julia; Notz, Dirk; Peters-von Gehlen, Karsten; Pincus, Robert; Pohlmann, Holger; Pongratz, Julia; Rast, Sebastian; Schmidt, Hauke; Schnur, Reiner; Schulzweida, Uwe; Six, Katharina; Stevens, Bjorn; Voigt, Aiko; Roeckner, Erich (2019). DKRZ MPI-ESM1.2-HR model output prepared for CMIP6 ScenarioMIP SSP5-8.5. Earth System Grid Federation. doi: https://doi.org/10.22033/ESGF/CMIP6.4403.
EC-Earth3	SSP1-2.6	EC-Earth Consortium (EC-Earth) (2019). EC-Earth-Consortium EC-Earth3 model output prepared for CMIP6 ScenarioMIP SSP1-2.6. Earth System Grid Federation. doi: https://doi.org/10.22033/ESGF/CMIP6.4874.
EC-Earth3	SSP2-4.5	EC-Earth Consortium (EC-Earth) (2019). EC-Earth-Consortium EC-Earth3 model output prepared for CMIP6 ScenarioMIP SSP2-4.5. Earth System Grid Federation. doi: https://doi.org/10.22033/ESGF/CMIP6.4880.

EC-Earth3	SSP5-8.5	EC-Earth Consortium (EC-Earth) (2019). EC-Earth-Consortium EC-Earth3 model output prepared for CMIP6 ScenarioMIP SSP5-8.5. Earth System Grid Federation. doi: https://doi.org/10.22033/ESGF/CMIP6.4912.
IPSL-CM6A-LR	SSP1-2.6	Boucher, Olivier; Denvil, Sébastien; Levavasseur, Guillaume; Cozic, Anne; Caubel, Arnaud; Foujols, Marie-Alice; Meurdesoif, Yann; Cadule, Patricia; Devilliers, Marion; Dupont, Eliott; Lurton, Thibaut (2019). IPSL IPSL-CM6A-LR model output prepared for CMIP6 ScenarioMIP SSP1-2.6. Earth System Grid Federation. doi: https://doi.org/10.22033/ESGF/CMIP6.5262.
IPSL-CM6A-LR	SSP2-4.5	Boucher, Olivier; Denvil, Sébastien; Levavasseur, Guillaume; Cozic, Anne; Caubel, Arnaud; Foujols, Marie-Alice; Meurdesoif, Yann; Cadule, Patricia; Devilliers, Marion; Dupont, Eliott; Lurton, Thibaut (2019). IPSL IPSL-CM6A-LR model output prepared for CMIP6 ScenarioMIP SSP2-4.5. Earth System Grid Federation. doi: https://doi.org/10.22033/ESGF/CMIP6.5264.
IPSL-CM6A-LR	SSP5-8.5	Boucher, Olivier; Denvil, Sébastien; Levavasseur, Guillaume; Cozic, Anne; Caubel, Arnaud; Foujols, Marie-Alice; Meurdesoif, Yann; Cadule, Patricia; Devilliers, Marion; Dupont, Eliott; Lurton, Thibaut (2019). IPSL IPSL-CM6A-LR model output prepared for CMIP6 ScenarioMIP SSP5-8.5. Earth System Grid Federation. doi: https://doi.org/10.22033/ESGF/CMIP6.5271.
MIROC6	SSP1-2.6	Shiogama, Hideo; Abe, Manabu; Tatebe, Hiroaki (2019). MIROC MIROC6 model output prepared for CMIP6 ScenarioMIP SSP1-2.6. Earth System Grid Federation. doi: https://doi.org/10.22033/ESGF/CMIP6.5743.
MIROC6	SSP2-4.5	Shiogama, Hideo; Abe, Manabu; Tatebe, Hiroaki (2019). MIROC MIROC6 model output prepared for CMIP6 ScenarioMIP SSP2-4.5. Earth System Grid Federation. doi: https://doi.org/10.22033/ESGF/CMIP6.5746.
MIROC6	SSP5-8.5	Shiogama, Hideo; Abe, Manabu; Tatebe, Hiroaki (2019). MIROC MIROC6 model output prepared for CMIP6 ScenarioMIP SSP5-8.5. Earth System Grid Federation. doi: https://doi.org/10.22033/ESGF/CMIP6.5771.
UKESM1-0-LL	SSP1-2.6	Good, Peter; Sellar, Alistair; Tang, Yongming; Rumbold, Steve; Ellis, Rich; Kelley, Douglas; Kuhlbrodt, Till (2019). MOHC UKESM1.0-LL model output prepared for CMIP6 ScenarioMIP SSP1-2.6. Earth System Grid Federation. doi: https://doi.org/10.22033/ESGF/CMIP6.6333.
UKESM1-0-LL	SSP2-4.5	Good, Peter; Sellar, Alistair; Tang, Yongming; Rumbold, Steve; Ellis, Rich; Kelley, Douglas; Kuhlbrodt, Till (2019). MOHC UKESM1.0-LL model output prepared for CMIP6 ScenarioMIP SSP2-4.5. Earth System Grid Federation. doi: https://doi.org/10.22033/ESGF/CMIP6.6339.
UKESM1-0-LL	SSP5-8.5	Good, Peter; Sellar, Alistair; Tang, Yongming; Rumbold, Steve; Ellis, Rich; Kelley, Douglas; Kuhlbrodt, Till (2019). MOHC UKESM1.0-LL model output prepared for CMIP6 ScenarioMIP SSP5-8.5. Earth System Grid Federation. doi: https://doi.org/10.22033/ESGF/CMIP6.6405.

MRI-ESM2-0	SSP1-2.6	Yukimoto, Seiji; Koshiro, Tsuyoshi; Kawai, Hideaki; Oshima, Naga; Yoshida, Kohei; Urakawa, Shogo; Tsujino, Hiroyuki; Deushi, Makoto; Tanaka, Taichu; Hosaka, Masahiro; Yoshimura, Hiromasa; Shindo, Eiki; Mizuta, Ryo; Ishii, Masayoshi; Obata, Atsushi; Adachi, Yukimasa (2019). MRI MRI-ESM2.0 model output prepared for CMIP6 ScenarioMIP SSP1-2.6. Earth System Grid Federation. doi: https://doi.org/10.22033/ESGF/CMIP6.6909.
MRI-ESM2-0	SSP2-4.5	Yukimoto, Seiji; Koshiro, Tsuyoshi; Kawai, Hideaki; Oshima, Naga; Yoshida, Kohei; Urakawa, Shogo; Tsujino, Hiroyuki; Deushi, Makoto; Tanaka, Taichu; Hosaka, Masahiro; Yoshimura, Hiromasa; Shindo, Eiki; Mizuta, Ryo; Ishii, Masayoshi; Obata, Atsushi; Adachi, Yukimasa (2019). MRI MRI-ESM2.0 model output prepared for CMIP6 ScenarioMIP SSP2-4.5. Earth System Grid Federation. doi: https://doi.org/10.22033/ESGF/CMIP6.6910.
MRI-ESM2-0	SSP5-8.5	Yukimoto, Seiji; Koshiro, Tsuyoshi; Kawai, Hideaki; Oshima, Naga; Yoshida, Kohei; Urakawa, Shogo; Tsujino, Hiroyuki; Deushi, Makoto; Tanaka, Taichu; Hosaka, Masahiro; Yoshimura, Hiromasa; Shindo, Eiki; Mizuta, Ryo; Ishii, Masayoshi; Obata, Atsushi; Adachi, Yukimasa (2019). MRI MRI-ESM2.0 model output prepared for CMIP6 ScenarioMIP SSP5-8.5. Earth System Grid Federation. doi: https://doi.org/10.22033/ESGF/CMIP6.6929.
