# Supplementary material for: Temporal dynamics of climate change exposure and opportunities for global marine biodiversity
Source: Nat Commun. 2024 Jul 15;15:5836. doi: 10.1038/s41467-024-49736-6 (PMC11251284; doi:10.1038/s41467-024-49736-6)
Supplement: Supplementary file 3 — Reporting Summary [file 41467_2024_49736_MOESM3_ESM.pdf]

nature portfolio

Corresponding author(s): Andreas Schwarz Meyer
Christopher Trisos

Last updated by author(s): Jun 13, 2024

Reporting Summary

Nature Portfolio wishes to improve the reproducibility of the work that we publish. This form provides structure for consistency and transparency in reporting. For further information on Nature Portfolio policies, see our Editorial Policies and the Editorial Policy Checklist.

Statistics

For all statistical analyses, confirm that the following items are present in the figure legend, table legend, main text, or Methods section.

n/a	Confirmed	
☐	☒	The exact sample size (n) for each experimental group/condition, given as a discrete number and unit of measurement
☒	☐	A statement on whether measurements were taken from distinct samples or whether the same sample was measured repeatedly
☒	☐	The statistical test(s) used AND whether they are one- or two-sided<br>*Only common tests should be described solely by name; describe more complex techniques in the Methods section.*
☐	☒	A description of all covariates tested
☒	☐	A description of any assumptions or corrections, such as tests of normality and adjustment for multiple comparisons
☐	☒	A full description of the statistical parameters including central tendency (e.g. means) or other basic estimates (e.g. regression coefficient) AND variation (e.g. standard deviation) or associated estimates of uncertainty (e.g. confidence intervals)
☒	☐	For null hypothesis testing, the test statistic (e.g. F, t, r) with confidence intervals, effect sizes, degrees of freedom and P value noted<br>*Give P values as exact values whenever suitable.*
☒	☐	For Bayesian analysis, information on the choice of priors and Markov chain Monte Carlo settings
☒	☐	For hierarchical and complex designs, identification of the appropriate level for tests and full reporting of outcomes
☒	☐	Estimates of effect sizes (e.g. Cohen's d, Pearson's r), indicating how they were calculated

Our web collection on statistics for biologists contains articles on many of the points above.

Software and code

Policy information about availability of computer code

Data collection	No software was used in the data collection process.
Data analysis	Data analysis was performed in R v 4.3.3 or higher. Computer code used in the analysis is available on Figshare (link shared in the manuscript).

For manuscripts utilizing custom algorithms or software that are central to the research but not yet described in published literature, software must be made available to editors and reviewers. We strongly encourage code deposition in a community repository (e.g. GitHub). See the Nature Portfolio guidelines for submitting code & software for further information.

Data

Policy information about availability of data

All manuscripts must include a data availability statement. This statement should provide the following information, where applicable:

- Accession codes, unique identifiers, or web links for publicly available datasets
- A description of any restrictions on data availability
- For clinical datasets or third party data, please ensure that the statement adheres to our policy

Species distribution data was obtained from the AquaMaps database (https://aquamaps.org). AquaMaps data is freely available to download for individual species. To use the full AquaMaps dataset, AquaMap's team should be contacted (info.aquamaps@gmail.com). Species distribution maps presented in this study have been included with permission from AquaMaps team. Sea surface temperature projections for SSPs 1-2.6, 2-4.5, and 5-8.5 for the Coupled Model Intercomparison Project 6 (CMIP6) are available from https://esgf-index1.ceda.ac.uk/search/cmip6-ceda/.

Research involving human participants, their data, or biological material

Policy information about studies with human participants or human data. See also policy information about sex, gender (identity/presentation), and sexual orientation and race, ethnicity and racism.

Reporting on sex and gender	*Use the terms sex (biological attribute) and gender (shaped by social and cultural circumstances) carefully in order to avoid confusing both terms. Indicate if findings apply to only one sex or gender; describe whether sex and gender were considered in study design; whether sex and/or gender was determined based on self-reporting or assigned and methods used. Provide in the source data disaggregated sex and gender data, where this information has been collected, and if consent has been obtained for sharing of individual-level data; provide overall numbers in this Reporting Summary. Please state if this information has not been collected. Report sex- and gender-based analyses where performed, justify reasons for lack of sex- and gender-based analysis.*
Reporting on race, ethnicity, or other socially relevant groupings	*Please specify the socially constructed or socially relevant categorization variable(s) used in your manuscript and explain why they were used. Please note that such variables should not be used as proxies for other socially constructed/relevant variables (for example, race or ethnicity should not be used as a proxy for socioeconomic status). Provide clear definitions of the relevant terms used, how they were provided (by the participants/respondents, the researchers, or third parties), and the method(s) used to classify people into the different categories (e.g. self-report, census or administrative data, social media data, etc.) Please provide details about how you controlled for confounding variables in your analyses.*
Population characteristics	*Describe the covariate-relevant population characteristics of the human research participants (e.g. age, genotypic information, past and current diagnosis and treatment categories). If you filled out the behavioural & social sciences study design questions and have nothing to add here, write "See above."*
Recruitment	*Describe how participants were recruited. Outline any potential self-selection bias or other biases that may be present and how these are likely to impact results.*
Ethics oversight	*Identify the organization(s) that approved the study protocol.*

Note that full information on the approval of the study protocol must also be provided in the manuscript.

Field-specific reporting

Please select the one below that is the best fit for your research. If you are not sure, read the appropriate sections before making your selection.

☐ Life sciences ☐ Behavioural & social sciences ☒ Ecological, evolutionary & environmental sciences

For a reference copy of the document with all sections, see nature.com/documents/nr-reporting-summary-flat.pdf

Ecological, evolutionary & environmental sciences study design

All studies must disclose on these points even when the disclosure is negative.

Study description	We combined future climate simulations with species distribution data and realised thermal niche data to project where and when (1) marine biodiversity are projected to be exposed to conditions beyond their thermal niche (thermal exposure), and (2) where and when new thermally suitable habitats are projected to arise arise (thermal opportunity).
Research sample	We used geographic distribution data for 21,696 marine taxa from 33 phyla. This sample reflects the data availability from AquaMaps (https://aquamaps.org), the most comprehensive database on the distribution of marine biodiversity.
Sampling strategy	The sample size reflects availability of geographic range data for marine taxa.
Data collection	Climate data was obtained from a public data portal. Geographic distribution data were provided by the AquaMaps team.
Timing and spatial scale	No temporal scale was used to obtain data for this study. AquaMaps distribution data reflects current environmental conditions (2000-2014). Analyses were carried out at a global-scale, with a spatial resolution of ~100 km.
Data exclusions	AquaMaps generates model-based range maps for marine taxa. The database contains distribution predictions for over 33,500 marine taxa. To avoid issues from data-scarce species, we excluded species for which models were generated using less than 10 occurrence records. We also excluded from the analyses species that occur exclusively below 200 m depth, as they are less likely to respond to changes in sea surface temperature. Finally, to ensure that realised thermal niche estimates are not affected by outliers, we excluded values that lay outside the range of ± 3 standard deviations from the maximum and minimum annual mean sea surface temperature experienced by each species across its entire range.
Reproducibility	This is not an experimental study so experimental replication was not attempted. All data used in our analysis can be obtained from public sources cited in the paper. The link to obtain the analysis code is share in the manuscript.
Randomization	No randomization was required.

Blinding | As our study was non-experimental, blinding is not applicable.

Did the study involve field work? ☐ Yes ☒ No

Reporting for specific materials, systems and methods

We require information from authors about some types of materials, experimental systems and methods used in many studies. Here, indicate whether each material, system or method listed is relevant to your study. If you are not sure if a list item applies to your research, read the appropriate section before selecting a response.

Materials & experimental systems

n/a	Involved in the study	
☒	☐	Antibodies
☒	☐	Eukaryotic cell lines
☒	☐	Palaeontology and archaeology
☒	☐	Animals and other organisms
☒	☐	Clinical data
☒	☐	Dual use research of concern
☒	☐	Plants

Methods

n/a	Involved in the study	
☒	☐	ChIP-seq
☒	☐	Flow cytometry
☒	☐	MRI-based neuroimaging
